# Predicting the efficiency of luminescent solar concentrators for solar energy harvesting using machine learning

**DOI:** 10.1038/s41598-024-54657-x

**Published:** 2024-02-20

**Authors:** Rute A. S. Ferreira, Sandra F. H. Correia, Lianshe Fu, Petia Georgieva, Mario Antunes, Paulo S. André

**Affiliations:** 1 CICECO-Aveiro Institute of Materials, Physics Department, University of Aveiro, 3810-193 Aveiro, Portugal; 2 Instituto de Telecomunicações, University of Aveiro, 3810-193 Aveiro, Portugal; 3 Department of Electronics Telecommunications and Informatics, University of Aveiro, 3810-193 Aveiro, Portugal; 4 Institute of Electronics and Informatics Engineering of Aveiro (IEETA), 3800-193 Aveiro, Portugal; 5 Department of Electrical and Computer Engineering and Instituto de Telecomunicações, Instituto Superior Técnico, Universidade de Lisboa, 1049-001 Lisbon, Portugal

**Keywords:** Luminescent solar concentrators, Machine learning, Clustering, Regression, Materials science, Optics and photonics

## Abstract

Building-integrated photovoltaics (BIPV) is an emerging technology in the solar energy field. It involves using luminescent solar concentrators to convert traditional windows into energy generators by utilizing light harvesting and conversion materials. This study investigates the application of machine learning (ML) to advance the fundamental understanding of optical material design. By leveraging accessible photoluminescent measurements, ML models estimate optical properties, streamlining the process of developing novel materials, offering a cost-effective and efficient alternative to traditional methods, and facilitating the selection of competitive materials. Regression and clustering methods were used to estimate the optical conversion efficiency and power conversion efficiency. The regression models achieved a Mean Absolute Error (MAE) of 10%, which demonstrates accuracy within a 10% range of possible values. Both regression and clustering models showed high agreement, with a minimal MAE of 7%, highlighting the efficacy of ML in predicting optical properties of luminescent materials for BIPV.

## Introduction

The need for the prediction of crystal structures based on chemical or material composition from produced or published experimental data is still a huge challenge^1,2^. Traditional methods of discovering new materials or materials with new properties, such as laboratory experiments have long development cycles and high costs^3^. Machine learning (ML) algorithms are a new promising tool to tackle this issue, opening the possibility of managing and extracting valuable insights from vast amounts of data^4^. The application of ML to materials science is recent^5^, with an emphasis on basic models in nature, being also possible to use ML as a simple fitting procedure for small low-dimensional datasets^1^.

Over the past decade, the progress of ML has greatly impacted the entire spectrum of physical sciences, including materials science^6^ and a reflection of that is the creation of the material genome initiative^7^ as a path for accelerating materials development and rationally designing materials through the use of data-driven methods. Other works report the use of ML linear regression methods to build force constant models for thermodynamic properties of materials used in physics and chemistry^8^ and the data-centric approach was used for polymer crystals^9^, pharmaceutical science^10^ and material constitutive modeling for metal forming processes^11^, with likely many more important developments to come shortly. Also, the importance of the increasing quantity of data recently collected (from experiments and simulations), for the advancement of the ML as a tool for phosphor design has already been discussed^12^. ML is thus poised to further enhance its role in materials science by playing a crucial role in identifying upcoming opportunities and challenges within this rapidly growing field^13^.

In an era overshadowed by climate change, environmental degradation, and the recent energy crisis, the fields of materials science and engineering have gained significant relevance. These fields offer crucial avenues to mitigate the repercussions of these challenges through the innovative capacity of materials, enabling them to predict, control, and enhance not only the behavior of materials but also their sustainable manufacturing, usage, and recyclability^14^. Photonics emerges as one of the main enabling technologies in the twenty-first century due to its fulminant growth and its potential to increase innovation in several industries such as photovoltaics that stands out recently in the field of building integrated photovoltaics to cope with energy needs and *in-situ* energy generation in buildings^15,16^.

**Figure 1:**
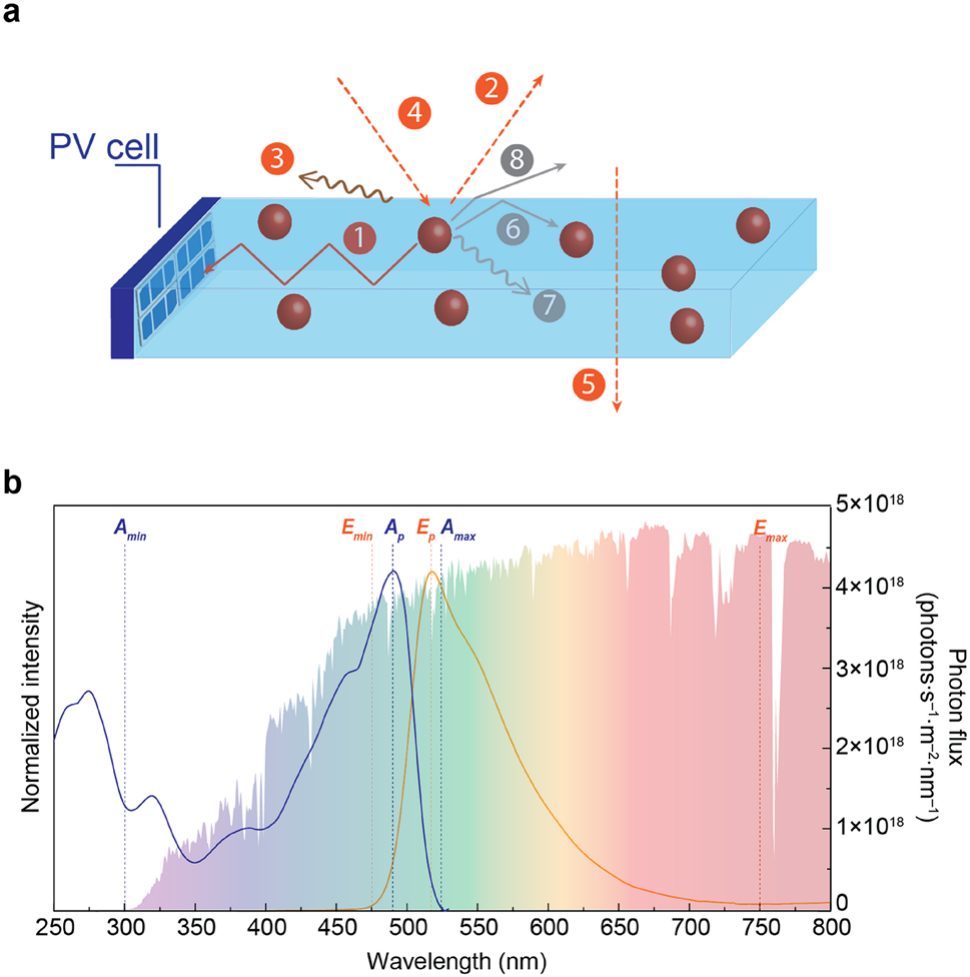
Luminescent solar concentrators and photoluminescence features. (**a**) Schematic representation of operating principles of planar LSCs: (1) emission from the optically active center, (2) Fresnel reflections, (3) surface scattering, (4) waveguide attenuation, (5) transmitted radiation, (6) re-absorption by neighbor centers, (7) non-radiative relaxation, (8) emission within the escape cone. (**b**) Excitation (blue line) and emission (orange line) spectra of luminescent carbon dots^20^ in aqueous solution monitored at 550 nm and excited at 360 nm, respectively. The shadowed area represents the AM1.5G solar spectrum photon flux (right *y* axis); the absorption peak (*A*_*p*_), the peak emission wavelength (*E*_*p*_), the minimum/maximum absorption wavelengths (*A*_*min*_/*A*_*max*_), and the minimum/maximum emission wavelengths (*E*_*min*_/*E*_*max*_) are also indicated. Adapted with permission under the terms of the Creative Commons Attribution License (https://creativecommons.org/licenses/by/3.0/)^20^. The *A*_*min*_ was set as 300 nm because below this the solar irradiance is very low (∼10^−4^% of the total solar irradiance on Earth) and *E*_*max*_ corresponds to the high-wavelength value of the emission spectrum, where the intensity exhibits significant deviation from the noise level (> 5%)^24,25^.

Luminescent solar concentrators (LSCs)^17,18^ represent a promising strategy for converting passive glass windows into self-sustaining energy sources^15^. By harnessing solar energy and transforming it into low-energy photons capable of generating electricity in solar cells, LSCs offer a revolutionary approach to energy generation (Fig. 1a)^15^. Essentially, an LSC comprises a waveguide—either planar or fiber—infused with optically active centers. These centers absorb the solar photons and subsequently convert it into low-energy photons. Guided by total internal reflection, these photons are directed towards photovoltaic cells positioned along the edges of the waveguide and efficiently converted into electricity. Notably, LSCs enable the utilization of solar radiation through large-area devices, utilizing minimal photovoltaic material—only required at the window edges. Electrical output values of 10 W per window are envisaged for a surface area of 0.05 to 0.1 m^2^ with the current figures of merit, granting, for instance, the power of Internet of Things (IoT) devices^19–21^. This opened the door to the consideration of larger windows, typical for residential buildings, with dimensions on the order of square meters, which could be conceptualized as aggregates of LSC with smaller dimensions without compromising the electrical output.

The optimization of the LSC performance is a complex task as its operating principles account for multiple events, such as emission from the optically active center, Fresnel reflections, surface scattering, waveguide attenuation, transmitted radiation, re-absorption by neighbor centers, non-radiative relaxation, and emission within the escape cone^15^ (Fig. 1a). Challenges include the design of spectral converters able to shape the sunlight to cope with the mismatch between the solar irradiance on Earth and the photovoltaic cells’ absorption since typical low-cost silicon solar cell presents low performance in the UV spectral region. Moreover, for application in facades, LSCs must ensure that the visible component of radiation is not absorbed to maintain transparency, redirecting the remaining components to the spectral region aligned with the PV's maximum absorption. Thus, we propose to use ML algorithms for data-driven research in the field of optical materials for photon management with an emphasis on low-power excitation, namely diffuse sunlight conditions such as cloudy days, where the relative intensity of UV photons related to that of the visible/NIR is higher than that found in clear sky conditions^22^.

The objective here is to discuss the use of ML as a valuable resource for decision-making tools for device design without extensive experimental measurements, as recently sugested^23^. A key step is the identification of the photoluminescence figures-of-merit related to performance (e.g. absorption and emission spectral ranges, quantum yield, photostability) that enable the outcomes (e.g. families of materials, concentration, preparation methods) to be properly benchmarked^24,25^. The independent identification will permit to impact on fundamental research, as typically stability and optical performance are addressed by distinct research fields, whereas engineers and industry can combine both simultaneously, as the commercial application requires stability and high performance.

We propose the use of classical ML-based regression and clustering methods as relevant fitting procedures for the estimation of underlying optical properties of luminescent materials. It is demonstrated that the η_opt_, and the power conversion efficiency (PCE) can be estimated from easily accessible, measurable optical features (i.e. absorption and emission spectra). The comparative study of the five most typically applied ML regression models, namely Gradient Boosting Regressor (GBR), Linear Regression, K- Nearest Neighbors Regressor, Random Forest Regressor, and XGBoost, demonstrate similar performance. After cleaning and removal of data outliers, the performance of the proposed regressors was further improved, demonstrating the high potential of a data-driven approach for the estimation of optical properties of new materials even for a small-size data set^24,25^.

## Results and discussion

### Data records

**Figure 2:**
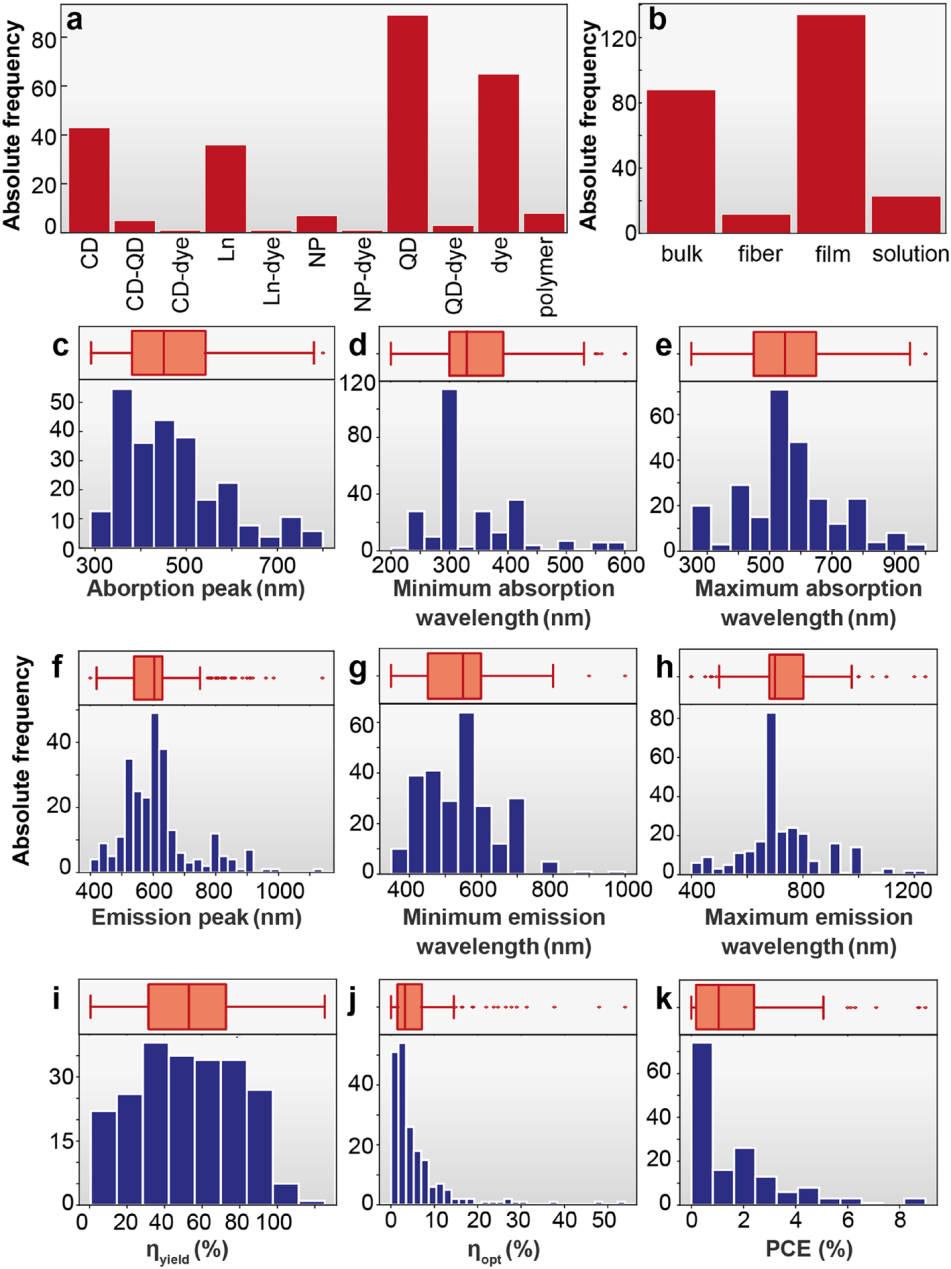
Data set—numerical and categorical features. Histograms and values distribution of (**a**) Type of the optical center (mat0), organic dye (dye), lanthanide ions (Ln), quantum dot (QD), carbon dot (CD), nanoparticle (NP); (**b**) processing feature (mat1), bulk, fiber, solution, film, (**c**) peak absorption wavelength (*A*_p_), (**d**) minimum absorption wavelength (A_min_), (**e**) maximum absorption wavelength (A_max_) (**f**) peak emission wavelength (*E*_p_) (**g**) minimum emission wavelength (*E*_min_), (**h**) maximum emission wavelength (*E*_max_), (**i**) absolute emission quantum yield (η_yield_), (**j**) optical conversion efficiency (η_opt_), and (**k**) power conversion efficiency (PCE).

The dataset consists of several numerical optical features of the first 260 entries of dataset available elsewhere,^24^ and shown in Fig. 2. The selected numerical features are the (i) peak absorption wavelength (*A*_*p*_), (ii) minimum absorption wavelength (*A*_*min*_), (iii) maximum absorption wavelength (*A*_*max*_) (iv) peak emission wavelength (*E*_*p*_) (v) minimum emission wavelength (*E*_*min*_), (vi) maximum emission wavelength (*E*_*max*_), and (vii) absolute emission quantum yield (*η*_*yield*_ defined as the ratio between the number of absorbed and emitted photons by a sample)^25^. To illustrate the physical meaning of each experimental parameter, Fig. 1b illustrates the absorption and emission spectra of a selected material. The parameters related with the absorption and emission bands are particularly important since they define the self-absorption, quantified by the overlap between the absorption and emission spectra which lead to re-absorption losses (e.g. large Stokes-shift^15,26^ defined for organic molecules). This has been pointed out as one of the most critical aspects for the device performance^15,16,27–31^, although its quantification is available in few works^25,27–29^. Photostability is also a crucial parameter for real application of LSC devices. Nevertheless, as this is often not reported in the published works and without standard conditions to be performed or reported, this was not included as a feature in this study.

Two categorical features (*mat0* and *mat1*) related to either the type of the optical center were also considered, namely organic dye (dye), lanthanide ions (Ln), quantum dot (QD), carbon dot (CD), nanoparticle (NP) or the processing feature (e.g. bulk, fiber, solution, film), respectively. The figures of merit that characterize the LSC performance are *η*_*opt*_, given by the ratio between the output optical power (*P*_*out*_) and the input optical power (*P*_*in*_) and PCE that accounts for the percentage of the solar energy incident on the LSC that is converted into usable electricity (Eq. S1-S4 in Supplementary Information). Table 1 shows a sample of the dataset illustrating the general optical features and the performance optical features for reported LSCs according to the optically active center.

**Table 1:** Sample of the table dataset^24,25^ showing the wavelength of peak absorption (*A*_*p*_), the minimum wavelength of the absorption spectral range (*A*_*min*_), the maximum wavelength of the absorption spectral range (*A*_*max*_), the wavelength of peak emission (*E*_*p*_), the minimum wavelength of the emission spectral range (*E*_*min*_), the maximum wavelength of the emission spectral range (*E*_*max*_), the quantum yield (***η***_***yield***_), optical conversion efficiency (***η***_***opt***_) and the power conversion efficiency (PCE) for reported LSCs according to the optically active center type and processing.

Optical center		General optical features							Performance optical features	
Type (mat0)	Processing (mat1)	Absorption			Emission					
		*A*_*p*_ (nm)	*A*_*min*_ (nm)	*A*_*max*_ (nm)	*E*_*p*_ (nm)	*E*_*min*_ (nm)	*E*_*max*_ (nm)	*η*_*yield*_ (%)	*η*_*opt*_ (%)	PCE (%)
Dye^36^	Film	770	300	800	775	700	950	16	1.5	–
Dye^21^	Bulk	665	250	700	670	600	750	12	3.7	0.10
Ln^37^	Fiber	370	300	450	615	570	710	89	0.7	–
Dye^37^		560	300	600	580	550	700	95	2.1	–
Dye^37^		780	300	750	730	650	850	21	0.5	–
Ln^37^		370	300	450	615	570	710	89	–	0.08
Dye^37^		560	300	600	580	550	700	95	–	0.21
QD^38^	Bulk	580	500	620	630	590	700	10	–	2.10
Dye^39^		498	300	700	580	520	700	30	6.88	0.27
Dye^40^		569	350	600	595	550	750	61	2.58	–
Dye^41^		488	300	500	510	450	600	51	3.3	0.35
CD^20^	Film	491	250	520	520	500	700	82	5.43	0.18
Dye^19^	Bulk	660	480	680	665	600	800	31	2.65	0.210

### Regression stage

**Figure 3:**
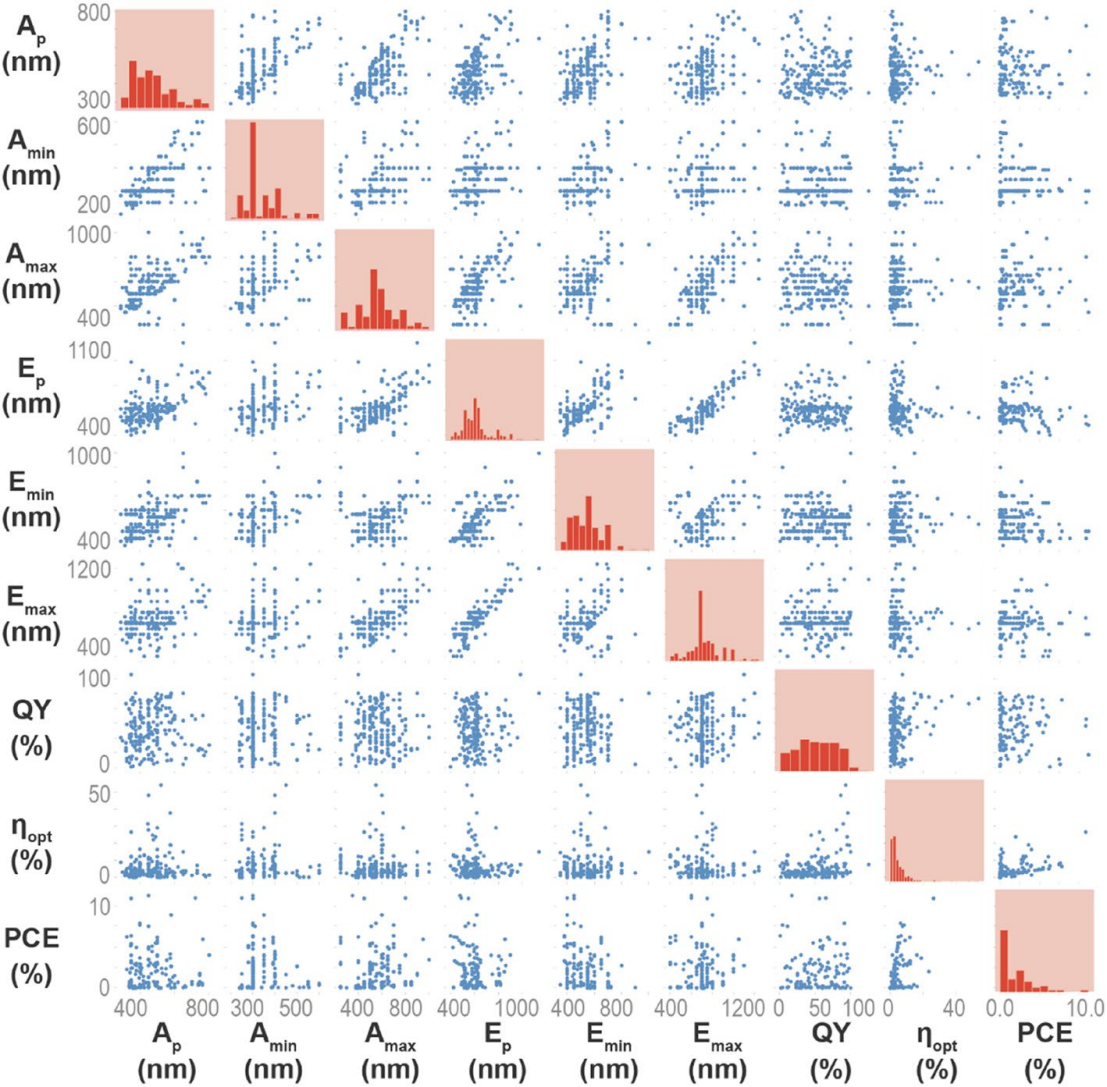
Features correlation. Pair-wise correlation between the six numerical input features and between them and the estimated outputs (PCE, *η*_opt_).

The analysis started with a quantitative assessment of the numerical distribution of each feature (Fig. 2) and a visual inspection of the mutual correlation between each pair of features and the correlation of the features with the estimated output variables (Figs. 3 and S1 in Supplementary Information), as detailed in the "Methods" section. Note that there is no substantial linear correlation between any of the input variables (the predictors) and the predicted outputs. A linear correlation was only observed between some of the features, namely *E*_*max*_ vs. *E*_*p*_ and *η*_*opt*_ vs. PCE (Fig. 3). The linear correlation between the optical features may be rationalized by attending to the typical Gaussian profile of the photoluminescence spectra, inducing the correlation between the *E*_*max*_ and *E*_*p*_ features^23^. The correlation between *η*_*opt*_ and PCE arises due to the fact that the larger number of incident photons are converted (quantified by *η*_*opt*_), the larger the probability to generate electrons in the photovoltaic cell (quantified by PCE). Noticeably, the PCE is mainly dependent on the semiconductor type used to fabricate the photovoltaic cell and on its efficiency^15^. As silicon-based photovoltaic cells were used in 90% of the data^25^, the optical response is similar, and therefore, PCE linearly correlates well with η_opt_.

We note that the performance in terms of *η*_*opt*_ is independent of the photovoltaic technology as it only quantifies the spectral conversion of the emitting layers, and thus this parameter can be predicted using ML algorithms, as recently shown using artificial neural networks^23^. Only the PCE values acquired with other semiconductor-based technologies (e.g. GaAs, Perovskite, CIGS—Copper Indium Gallium Selenide, CuInSe_2_, organic sensitive dye,) lie outside the linear correlation because those materials display a distinct responsivity compared with silicon. Nevertheless, it is shown that correlation between the real and predicted values is high even for the cases of the dataset where the photovoltaic technology is not silicon-based, proving that the algorithm will work also for these cases, considering the error.

Tables 2 and 3 list the estimated values for PCE and *η*_*opt*_, respectively, considering 6, 7, or 9 predictors (input variables) for the scenarios where the models were trained with all available data with the outliers, or after the removal of the outliers (see details in the "Methods" section). The error distribution, in the shape of violin plots for the 5 regression models (details in the "Methods" section) provided with different input features are represented in Figs. S2–S5 in Supplementary Information. In each of the scenarios, the data set used to fit the regression models vary because the samples with missing values were discarded. For example, in the baseline model with 6 input features (*A*_*p*_, *A*_*min*_, *A*_*max*_,* E*_*p*_, *E*_*min*_, *E*_*max*_), only the rows with available values for PCE or η_opt,_ were considered. For the scenarios of 7 (*A*_*p*_, *A*_*min*_, *A*_*max*_,* E*_*p*_, *E*_*min*_, *E*_*max*_, *η*_*yield*_) or 9 predictors (*A*_*p*_, *A*_*min*_, *A*_*max*_,* E*_*p*_, *E*_*min*_, *E*_*max*_, *η*_*yield*_, mat0, mat1), only rows with available *η*_*yield*_ and PCE or *η*_*opt*_ were taken. These constraints are due to the supervised approach of learning, where the models are fitted with complete input–output information.

**Table 2:** PCE estimation with regression models (results with the validation samples from the K-fold CV experiments).

Regression model	Outliers	Evaluation metrics								
		MAE			MSE			R2		
		Inputs								
		*6*^a^	*7*^b^	*9*^c^	*6*^a^	*7*^b^	*9*^c^	*6*^a^	*7*^b^	*9*^c^
Linear	Not removed	1.21	1.33	1.29	2.03	3.27	2.94	0.19	0.00	0.15
	Removed	0.98	1.04	0.87	1.84	1.56	1.44	0.12	0.30	0.20
K-nearest neighbors	Not removed	1.05	0.91	0.98	2.23	2.55	2.39	0.11	0.23	0.31
	Removed	0.72	0.82	0.74	1.13	1.10	1.27	0.46	0.24	0.30
Random forest	Not removed	0.94	0.91	1.03	1.66	2.02	2.43	0.34	0.39	0.29
	Removed	0.70	0.63	0.62	1.01	0.95	0.74	0.52	0.57	0.59
Gradient boosting	Not removed	1.08	0.93	1.03	2.45	2.56	2.60	0.02	0.22	0.24
	Removed	0.85	0.84	0.49	1.58	1.79	0.60	0.24	0.19	0.67
XGBoost	Not removed	1.01	1.01	1.23	1.56	2.55	3.20	0.38	0.22	0.07
	Removed	0.80	0.77	0.61	1.30	1.75	1.01	0.38	0.21	0.44

^a^6 input features (*A*_*p*_, *A*_*min*_, *A*_*max*_,* E*_*p*_, *E*_*min*_, *E*_*max*_), ^b^7 input features (*A*_*p*_, *A*_*min*_, *A*_*max*_,* E*_*p*_, *E*_*min*_, *E*_*max*_, *η*_*yield*_), ^c^all numerical and categorical features (*A*_*p*_, *A*_*min*_, *A*_*max*_,* E*_*p*_, *E*_*min*_, *E*_*max*_, *η*_*yield*_, mat0, mat1).

**Table 3:** *η*_*opt*_ estimation with regression models (results with the validation samples from the K-fold CV experiments).

Regression model	Outliers	Evaluation metrics								
		MAE			MSE			R2		
		Inputs								
		*6*^a^	*7*^b^	*9*^c^	*6*^a^	*7*^b^	*9*^c^	*6*^a^	*7*^b^	*9*^c^
Linear	Not removed	4.11	4.33	3.15	32.50	38.71	20.75	− 0.08	0.05	0.49
	Removed	1.46	1.25	1.16	2.81	2.32	2.13	− 0.02	0.03	0.11
K-nearest neighbors	Not removed	2.38	2.98	2.93	12.45	23.43	22.28	0.58	0.43	0.46
	Removed	1.12	0.98	1.11	2.12	1.64	1.95	0.23	0.31	0.18
Random forest	Not removed	4.20	3.47	4.43	32.08	29.89	42.65	− 0.07	0.27	− 0.04
	Removed	1.24	1.17	1.20	2.19	2.54	2.43	0.20	− 0.06	− 0.02
Gradient boosting	Not removed	3.43	3.63	4.41	22.79	35.46	52.49	0.24	0.13	− 0.28
	Removed	1.34	1.51	1.50	2.62	3.50	3.86	0.05	− 0.47	− 0.62
XGBoost	Not removed	3.66	3.29	5.00	31.29	33.77	58.79	− 0.04	0.17	− 0.44
	Removed	1.47	1.29	1.15	2.94	2.94	2.62	− 0.07	− 0.23	− 0.10

^a^6 input features (*A*_*p*_, *A*_*min*_, *A*_*max*_,* E*_*p*_, *E*_*min*_, *E*_*max*_), ^b^7 input features (*A*_*p*_, *A*_*min*_, *A*_*max*_,* E*_*p*_, *E*_*min*_, *E*_*max*_, *η*_*yield*_), ^c^all numerical and categorical features (*A*_*p*_, *A*_*min*_, *A*_*max*_,* E*_*p*_, *E*_*min*_, *E*_*max*_, *η*_*yield*_, mat0, mat1).

The error-related metrics mean absolute error (MAE) and mean square error (MSE), demonstrate that shallow learning models can estimate PCE and *η*_*opt*_ with a substantial level of accuracy, based only on the absorption and emission features (the scenario with 6 input variables). A marginal positive effect is observed when extra features such as *η*_*yiel*d_ and the characteristics of the materials (*mat0* and *mat1*) are also included. These results point out the performance is mainly determined by the absorption spectra (overlap with AM1.5) and the emission spectra (overlap with the photovoltaic cell operation range).

Except for the linear regression (the case without outlier removal), the other regression methods converge to similar performance metrics that leverage confidence in the obtained results. Since PCE values range in (0–10) intervals (see Fig. 2k), MAE around 1 or less than 1, for most of the scenarios, corresponds to a maximum 10% error in the estimation of PCE which is rather an acceptable result considering that the estimation is based on standard photoluminescent readily accessible measurements. Similar conclusions can be drawn regarding the *η*_*opt*_ ranging in (0–50) interval (see Fig. 2j), and MAE less than 4, in most of the cases. Though the models demonstrate to be equally competitive, the Random Forest (RF) regressor (with removed outliers) slightly outperforms the other models. Similar results were reported, using RF to estimate the data uncertainty^32^.

We conclude that training an ensemble of decision trees parallel models on different replicas of the data is favorable to outputting more robust estimation in the presence of data uncertainty. In all models, the estimation accuracy improves by removing the outliers (Figs. S2–S5 in Supplementary Information). However, it should be noted that removing the outliers may hinder the models' ability to estimate high levels of PCE and *η*_*opt*_ because they were truncated. Note that the results in Tables 2 and 3 are concerning the test samples from the validation (10% of the complete dataset) which is a relatively small number of samples. Therefore, the explained variances measured by the Coefficient of determination (R^2^) metrics do not exhibit high values.

### Clustering stage

**Figure 4:**
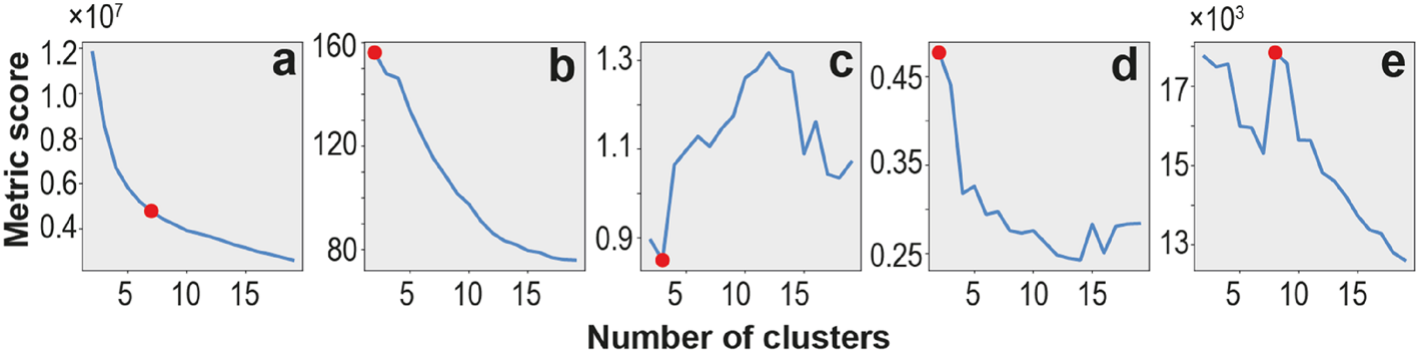
Clustering quality. Clustering quality metrics for (**a**) Elbow, (**b**) Calinski-Harabasz, (**c**) Davies-Bouldin, (**d**) Silhouette, and (**e**) BIC for different numbers of clusters (from 1 to 20 clusters). A red dot indicates the best number of clusters each metric suggests.

The K-Means clustering algorithm was used for further validation of the regression models. The goal is to separate the data samples into coherent clusters and estimate the missing PCE and *η*_*opt*_ from the available measurements inside each cluster (see “Methods” section for details). Figure 4 illustrates the lack of unequivocal agreement among various clustering metrics regarding the optimal number of clusters. However, five clusters seem an average between their suggestions and a relevant number for the current dataset size. Hence, K-Means is set to K = 5 clusters. In the first step, each sample is assigned to the closest cluster based on the minimum distance between the sample and K cluster centroids. Next, for the samples whose PCE or *η*_*opt*_ values are unknown, the *n* closest samples (in our experiments *n* = 3) with available PCE and *η*_*opt*_, were identified. The missing PCE and *η*_*opt*_ are then estimated as the median of the available measurements of the closest samples.

### Regression vs. clustering

The concordance between the cluster-based estimations (unsupervised approach) and the regression models (supervised approach) is evaluated. Though the regression models demonstrate similar performance, we selected the K-Nearest Neighbor to compare with the K-Means clustering, due to its simplicity, no need for loss function optimization, and low amount of hyperparameters to choose.

**Figure 5:**
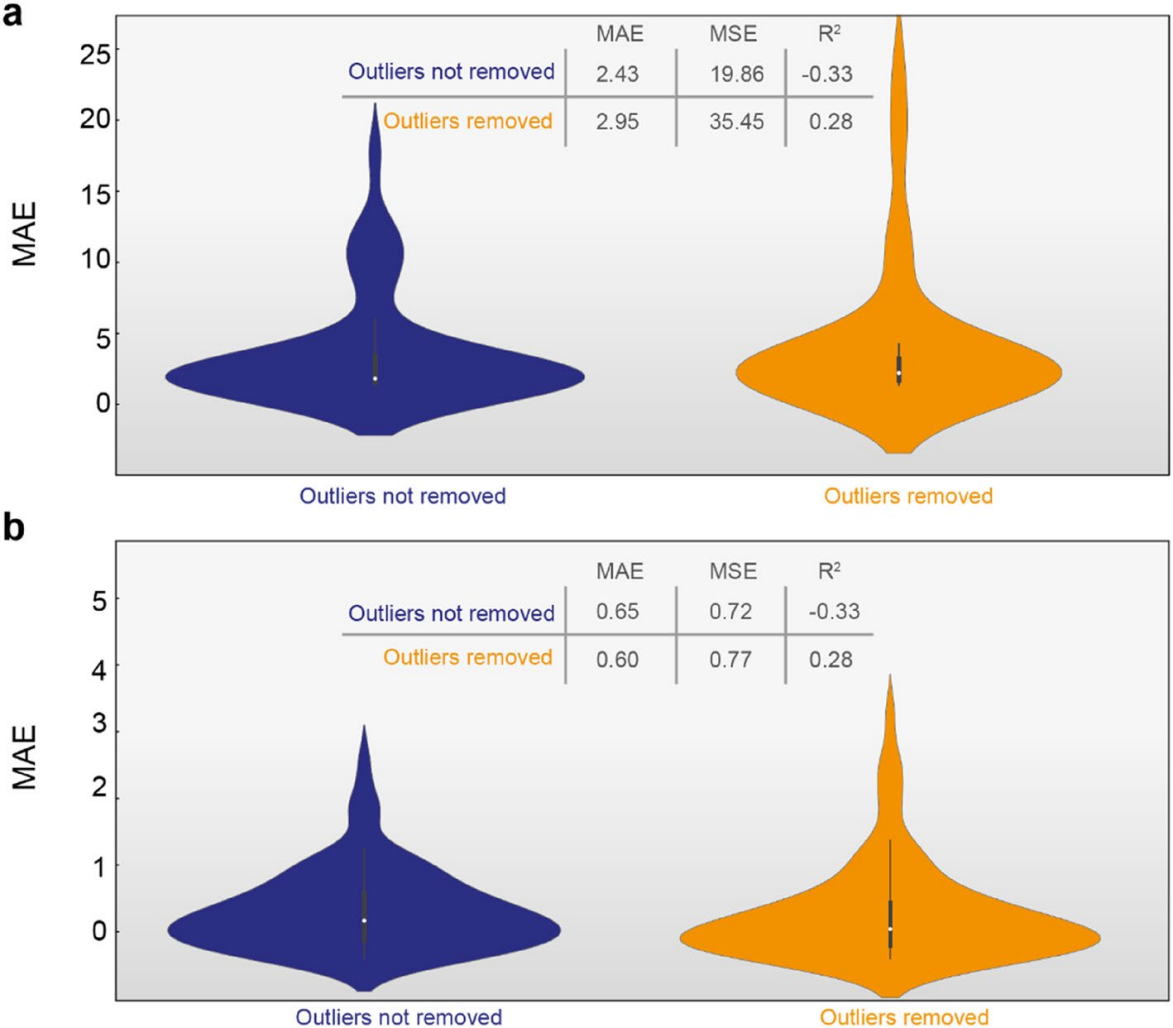
MAE violin plots for (**a**) η_opt_ and (**b**) PCE for the cluster-based estimation and the KNN regression. MSE and R^2^ metrics for the same scenarios (with and without removed outliers).

Figure 5 depicts the MAE violin plots representing the mean absolute error between the estimation of PCE and η_opt._ provided by the KNN regression models and the K-Means clustering. The MSE and R^2^ metrics for the same scenarios (with and without removed outliers) are also presented. Since PCE values range in (0–10) intervals (Fig. 2k), MAE equal to 0.65 (without removed outliers) or 0.60 (with removed outliers) means around 6% error between the K-means and the KNN estimations, which is overall an acceptable disagreement between the two approaches. Similarly, *η*_*opt*_ values are in the range of (0–50) interval (Fig. 2j), therefore MAE equal to 2.43 (without removed outliers) or 2.95 (with removed outliers) means less than 7% error which is also a very good agreement. Hence, the agreement between the two approaches (regression and clustering) is well demonstrated for the estimation of both PCE and *η*_*opt*_.

The presented results show that the regression models are naturally a more reliable approach to estimating the targeted quantities particularly when the dataset is relatively small. If the statistical variability of the estimated variable is limited and well covered by the data, clustering is a simple way to validate the regression models and can also be used as a viable alternative. Further to that, our findings suggest that it is always plausible to find a way to augment the data to enhance the generalization capacity of the models.

In this work, the potential of ML techniques to foster materials development and optimization is explored. The research question we pose is how to reliably estimate the optical performance of luminescent materials for BIPV using only easily available information such as conventional photoluminescence and knowledge about the optical active center. We propose two stages of ML-based rational design. During the first stage, the optical performance features (PCE and *η*_*opt*_) of the dataset of materials are estimated by applying regression models. At this stage, the photoluminescence measurements *A*_*p*_, *A*_*min*_, *A*_*max*_,* E*_*p*_, *E*_*min*_, and *E*_*max*_, are determined as the most suitable predictors. Our finding confirmed that once the most relevant features are properly identified, the choice of the ML model is less critical. The five regression methods exhibited similar performance that further enhanced the confidence in the proposed data-driven approach. The training and the validation of the regression models and the selection of their hyper-parameters followed the standard ML tuning process, namely K-fold cross-validation and the Grid search. In the second stage, we applied the K-means clustering technique to separate the materials into coherent groups and estimate the missing values for PCE and *η*_*opt*_ reinforcing the conclusions derived in the first stage. The major takeaway from this study is that the proposed rational design through the combination of supervised and unsupervised ML techniques is a promising way to speed up the development of new luminescent materials for sunlight harvesting and energy conversion. Combining the photoluminescence features used as inputs with the particularly versatile ML methods, the present work opens new perspectives for developing materials in a large variety of photonic applications beyond photovoltaics, where photoluminescence-related features are crucial (e.g. lighting and sensing).

## Methods

### Supervised learning with regression models

We aim to establish a reliable relationship between the experimental numerical features and the optical performance, therefore independent regression models were built for *η*_*opt*_ and PCE. The feature importance was also analyzed, and the most discriminative features were defined. Five regression models were constructed and tuned: Linear Regression, K-Nearest Neighbors, Random Forest, Gradient Boosting, and XGBoost (Supplementary Information for details).

The feature distribution in Fig. 2 shows the extreme values, which were not related to measurement errors, so they were considered outliers. Typically, the regression models are negatively influenced by the outliers, therefore two regression analyses were conducted building regression models with (i) all available values (without removal of outliers), and (ii) after filtering outliers using the interquartile range (IQR) method (Supplementary Information for details).

As the dataset contains 14%, 24%, and 41% of missing values for the optical properties *η*_*yield*_, *η*_*opt*,_ and PCE, respectively^25^, to establish a baseline, we assessed the predictability of PCE and *η*_*opt*_ using only the six absorption and emission features without missing values (*A*_*p*_, *A*_*min*_, *A*_*max*_,* E*_*p*_, *E*_*min*_, and *E*_*max*_). To cope with the limited size of the dataset, we employed shallow learning models, and the dataset was split into 90% and 10% for training and testing, respectively. The split was adapted for the regression problem, as it divides the target variable into bins and then uses them for the train-test stratification process. This ensures that the resulting split has a data distribution as close as possible to the original dataset, about the target variable. Grid Search was implemented to set up the optimal model hyperparameters and K-fold Cross Validation (CV) was used for the training subset (the K-fold CV was also adapted for regression problems). Since the estimated properties are continuous variables, we chose three evaluation metrics: MAE, MSE, and R^2^. The MAE measures the average magnitude of the errors in a set of predictions and is calculated as the average absolute difference between the actual values and the predicted values and MSE quantifies the average squared differences between predicted and actual values. The smaller the MAE or the MSE, the better the model is at predicting the outcome. Finally, R^2^ is a statistical measure that represents how well a regression model fits the data. It measures how much of the variation in the dependent variable can be explained by the independent variables^33–35^.

### Clustering approach

Clustering the dataset using only the six numerical input features (*A*_*p*_, *A*_*min*_, *A*_*max*_,* E*_*p*_, *E*_*min*_, *E*_*max*_) was used to analyze if there are patterns in these features that allow clustering the materials with enhanced optical performance (larger *η*_*opt*_ and PCE values).

The first step is to select the relevant number of clusters to understand the data. For that, we applied five different metrics to evaluate the cluster quality: Elbow, Calinski-Harabasz, Davies-Bouldin, Silhouette, and Bayesian information criterion (BIC). Each metric was computed from 1 to 20 clusters. The Elbow method is a heuristic approach to determine the most relevant number of clusters in a data set. It works by plotting the explained variation as a function of the number of clusters and picking the elbow of the curve as the number of clusters to use. The Calinski-Harabasz index rewards clustering in which the cluster centroids are far apart, and the cluster members are close to their respective centroids. The Davies-Bouldin index is defined as the average similarity measure of each cluster with its most similar cluster, where similarity is the ratio of within-cluster distances to between-cluster distances. The Silhouette score calculates how similar an object is to its cluster compared to other clusters. The Silhouette score ranges from − 1 to 1, where a score closer to 1 indicates that the object is well-matched to its cluster and poorly matched to neighboring clusters. The BIC is a metric for model selection among a finite set of models so that the model with the lowest BIC is preferred.

### Supplementary Information

Supplementary Information.

## Data Availability

The data that support the findings of this study are available at figShare (10.6084/m9.figshare.24903387.v1).

## Code availability

The code that supports the findings of this study is available at GitHub (https://github.com/mariolpantunes/sunlight-harvesting-energy-conversion).
