# Supplementary material for: Predicting the efficiency of luminescent solar concentrators for solar energy harvesting using machine learning
Source: Sci Rep. 2024 Feb 20;14:4160. doi: 10.1038/s41598-024-54657-x (PMC10879533; doi:10.1038/s41598-024-54657-x)
Supplement: Supplementary file 1 — Supplementary Information. [file 41598_2024_54657_MOESM1_ESM.docx]

SUPPORTING INFORMATION

Predicting the efficiency of luminescent solar concentrators for solar energy harvesting using machine learning

Rute A. S. Ferreira^1*^ Sandra F.H. Correia,^2^ Lianshe Fu,^1^ Petia Georgieva, ^2,3,4^ Mario Luis Pinto Antunes,^2,3^ Paulo S. André^5*^

Rute A. S. Ferreira^1*^ Sandra F.H. Correia,^2^ Lianshe Fu,^1^ Petia Georgieva,^2,3,4^ Mario Luis Pinto Antunes,^2,3^ Paulo S. André^5*^

^1^ CICECO-Aveiro Institute of Materials, Physics Department, University of Aveiro, 3810-193 Aveiro, Portugal.

^2^ Instituto de Telecomunicações, University of Aveiro, 3810-193 Aveiro, Portugal.

^3^ Department of Electronics Telecommunications and Informatics, University of Aveiro, 3810-193 Aveiro, Portugal.

^4^ Institute of Electronics and Informatics Engineering of Aveiro (IEETA), 3800-193 Aveiro, Portugal

^5^ Department of Electrical and Computer Engineering and Instituto de Telecomunicações, Instituto Superior Técnico, Universidade de Lisboa, 1049-001 Lisbon, Portugal

[*rferreira@ua.pt; paulo.andre@lx.it.pt](mailto:*rferreira@ua.pt;%20paulo.andre@lx.it.pt)

**S1. Methodology**

**S1.1 Figures of merit**

The figures of merit that characterize the LSC performance are the optical conversion efficiency (*η_opt_*) given by the ratio between the output optical power (*P_out_)* and the input optical power (*P_in_*). Regarding the experimental assessment of *η_opt_*, we note that different expressions are presented in the literature, as described in the following equations. The most often used quantification of *η_opt_* is through equations 1 and 2, namely:

| $\eta_{\mathrm{opt}}\text{=}\frac{\text{P}_{\text{out}}}{\text{P}_{\text{i}}}\text{=}\frac{\text{I}_{\text{SC}}^{\text{L}}\text{V}_{\text{0}}^{\text{L}}}{\text{I}_{\text{SC}}\text{V}_{\text{0}}}\frac{\text{A}_{\text{e}}}{\text{A}_{\text{s}}}\frac{\text{η}_{\text{solar}}}{\text{η}_{\text{PV}}}$ | (S1) |
| --- | --- |

where $I_{SC}^{L}$ and $V_{0}^{L}$ represent the short-circuit current and the open voltage when the PV device is coupled to the LSC ($I_{sc}$ and $V_{0}$ are the corresponding values of the PV device exposed directly to the solar radiation), $\eta_{solar}$ is the efficiency of the PV device relatively to the total solar spectrum and $\eta_{PV}$ is the efficiency of the PV device at the LSC emission wavelengths.^1^ An alternative definition is also often reported given by:^2^

| $\eta_{\mathrm{opt}}\text{=}\frac{\text{I}_{\text{SC}}^{\text{L}}}{\text{I}_{\text{SC}}}\frac{\text{A}_{\text{e}}}{\text{A}_{\text{s}}}$ | (S2) |
| --- | --- |

Another experimental approach used only in 6 cases,^3-7^ considers that *η_opt_* can be described by weighting all the losses in the LSC (Fig. 1B), given by the product of several terms:^8^

| $\eta_{opt}={\left( 1-R \right)\eta}_{abs}\eta_{SA}\eta_{yield}\eta_{Stokes}\eta_{trap}\eta_{mat}$ | (S3) |
| --- | --- |

in which *R* is the Fresnel reflection coefficient for perpendicular incidence, η*_abs_* is the ratio of photons absorbed by the emitting layer to the number of photons falling on it, *η_SA_* is the self-absorption efficiency, *η_Stokes_* is the Stokes efficiency and *η_tr_* takes into account the transport losses and *η_trap_*  is the trapping efficiency.

The PCE figure of merit is obtained from experimental data using the following equation (S4):

| $PCE=\frac{P_{out}^{el}}{P_{in}}=\frac{\text{I}_{\text{SC}}^{\text{L}}\text{V}_{\text{oc}}^{\text{L}}FF}{A_{S}\int_{\lambda_{1}}^{\lambda_{2}} I_{AM1.5G}\left( \lambda\right)d\lambda}$ | (S4) |
| --- | --- |

where $P_{out}^{el}$ and FF are the PV device output electrical power and fill factor of the PV cell, respectively.

**S1.2 Supervised learning with Regression Models**

The most widely applied shallow regression models were comparatively studied in the present work, to estimate the optical properties of the available dataset of luminescent materials. The models are shortly introduced below, more detailed information can be found elsewhere.^9-11^

**Linear Regression (LR)** is a statistical method for modeling the relationship between a dependent variable and one or more independent variables. It assumes a linear relationship between the independent variables (the model inputs) and the dependent variable (the model output). During the model training the parameters of the linear relation are fitted.

**K - Nearest Neighbors (k-NN)** is a classical non-parametric statistical technique used both for regression and classification purposes. For example, in k-NN regression, the value of the predicted target is computed as the average of the values of the K nearest neighbours. The K nearest neighbours are defined based on a similarity measure (such as Euclidian distance, Manhattan distance, etc.).

**Random forest (RF)** belongs to the ensemble learning algorithms, where a number of Decision Trees (DT) are trained independently, and their predictions are aggregated to identify the most popular result. The most well-known ensemble approaches are bagging and boosting. Typically, the bagging approach is applied in RF training, where data samples are randomly separated into bags and each DT is trained with a different portion of the data. RF is very successful in reducing the variance within a noisy dataset.

**Gradient Boosting Regressor (GBR)** is also an ensemble learning method, where multiple models are combined to improve the model performance. The main difference between Random Forests and Gradient Boosting lies in how the Decision Trees are created and aggregated. GBR follows a sequential training process, where at each stage one decision tree (DT) model is fitted and added to the ensemble. This process continues until a predefined number of DT regressors is fitted or until convergence is reached. GBR is known for its good capacity to handle complex non-linear relationships between the descriptors (features) and the predicted variable. In average GBR performs better than RF if parameters are tuned carefully, and this was confirmed in the present results.

**Extreme Gradient Boosting (XGBoost)** regressor is similar to GBR, however it has a more regularized model formalization (L1 & L2) to control over-fitting and improves model generalization capabilities. Its training can be parallelized across clusters and therefore in contrast to GBT it provides parallel tree boosting. XGBoost generally provides better accuracy compared to Gradient Boosting.

**S2. Results**

**
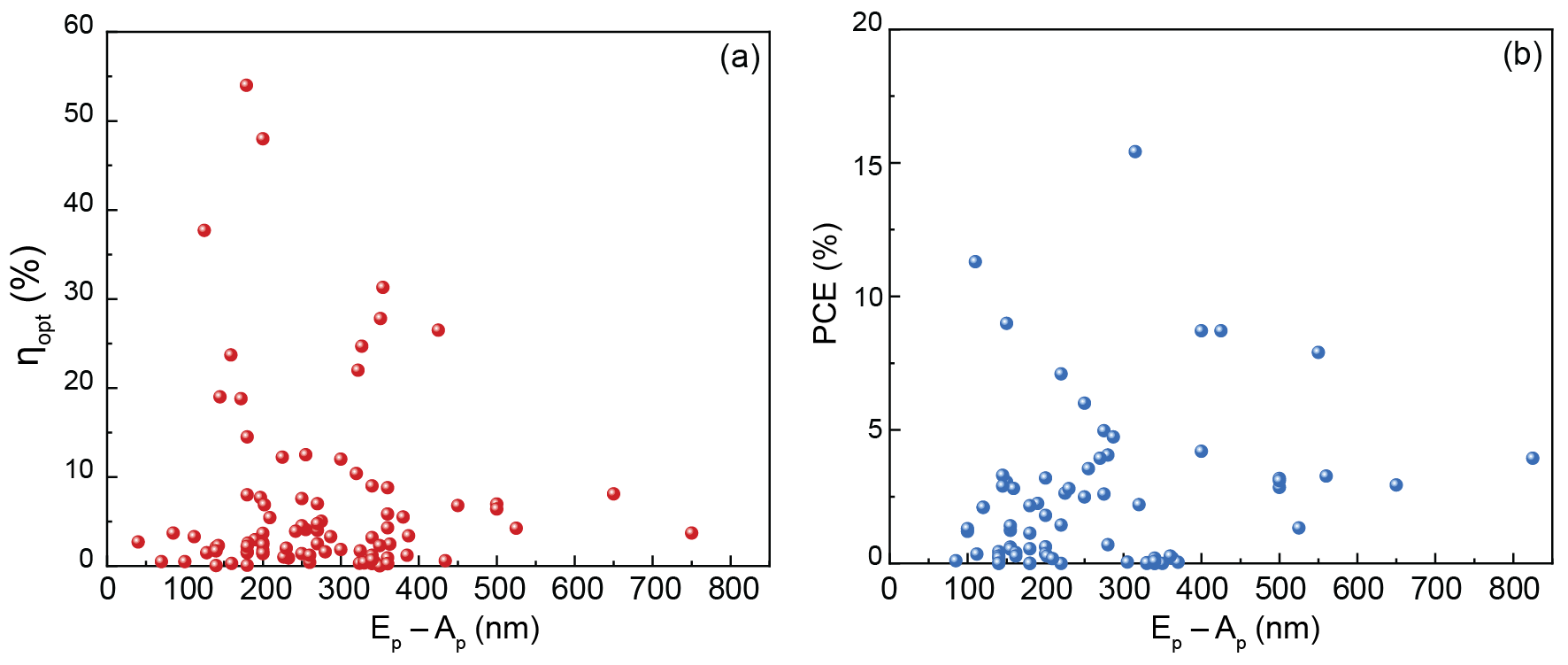
**

Fig. S1 Correlation between the reported (a) *η_opt_* and (b) PCE figures of merit and the spectral distance (measured in nm) between absorption and emission peak wavelengths.


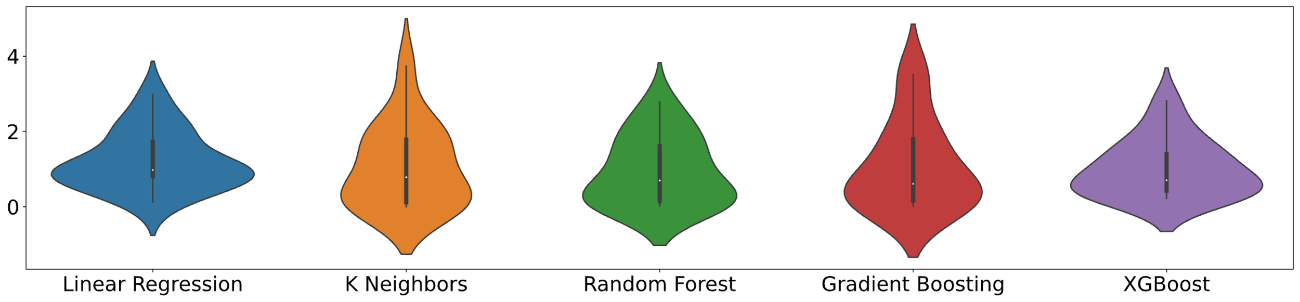


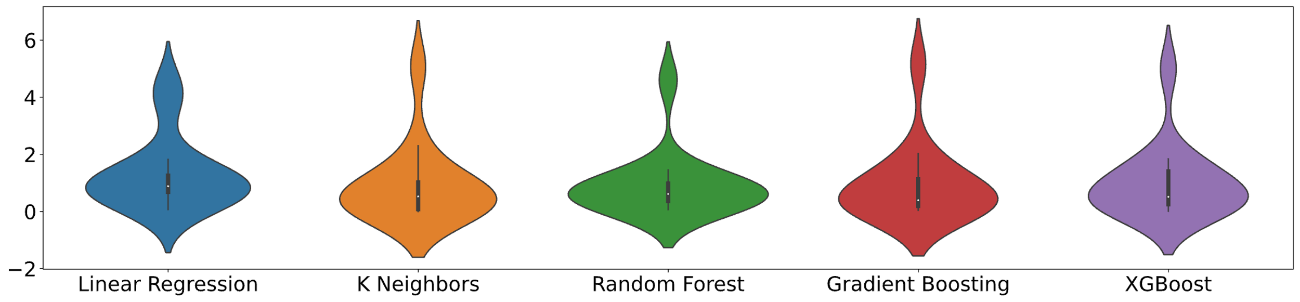


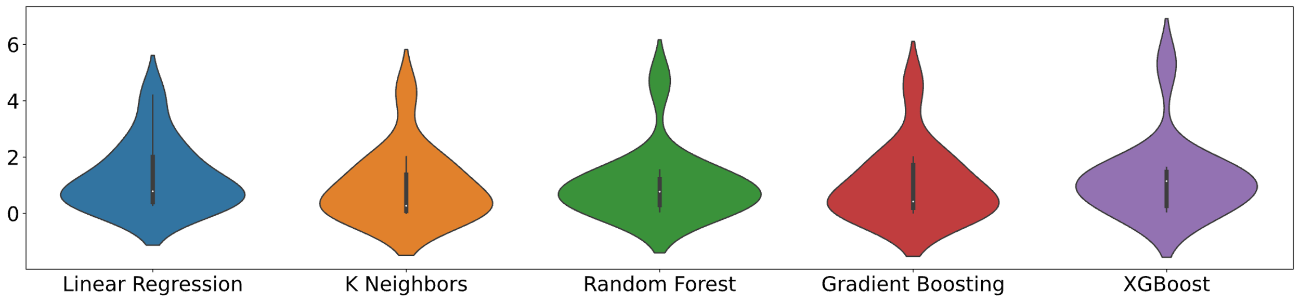


Fig. S2 PCE error distribution for 5 regression models (**all data with outliers**). Top (6 input features (*A_p_*, *A_min_*, *A_max_*, *E_p_*, *E_min_*, *E_max_*); Middle (7 input features (*A_p_*, *A_min_*, *A_max_*, *E_p_*, *E_min_*, *E_max_*, *η_yield_*); Bottom all numerical and categorical features (*A_p_*, *A_min_*, *A_max_*, *E_p_*, *E_min_*, *E_max_*, *η_yield_*, *mat0*, *mat1*).


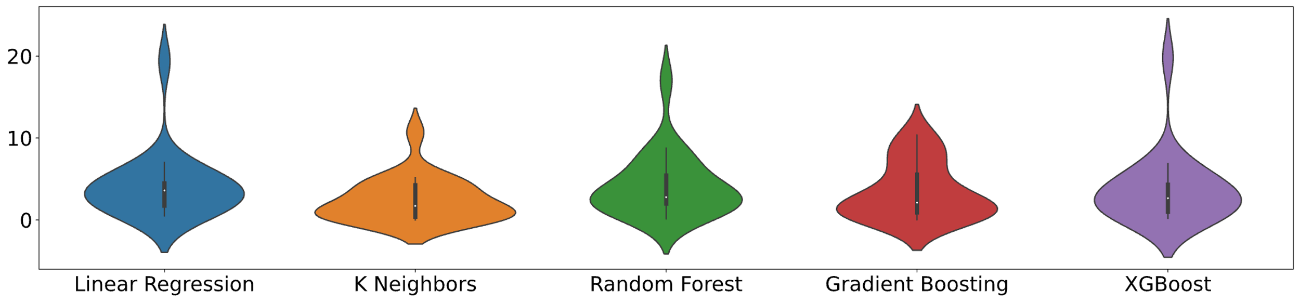


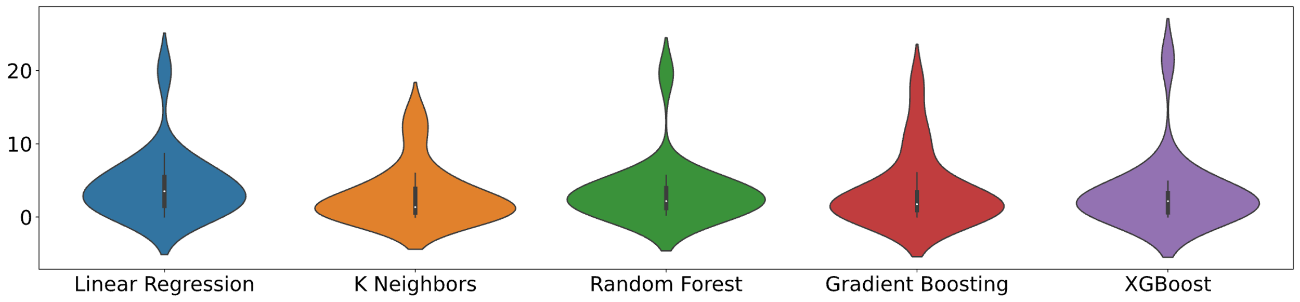


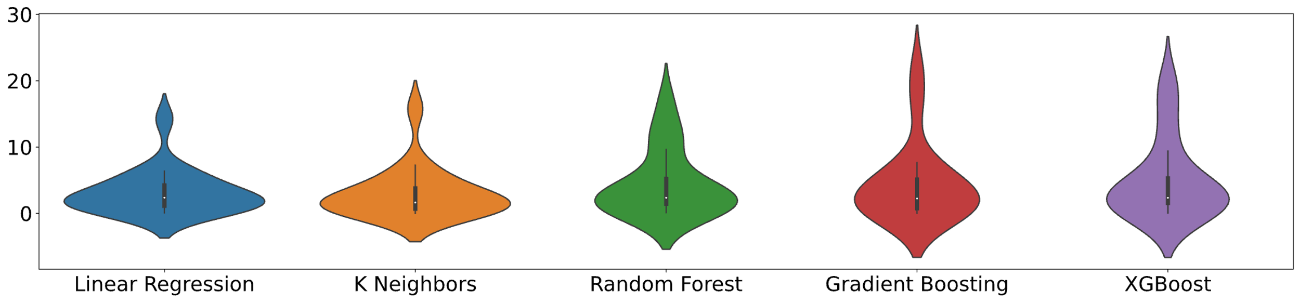


Fig. S3 η_opt_ error distribution for 5 regression models (**all data with outliers**). Top (6 input features (*A_p_*, *A_min_*, *A_max_*, *E_p_*, *E_min_*, *E_max_*); Middle (7 input features (*A_p_*, *A_min_*, *A_max_*, *E_p_*, *E_min_*, *E_max_*, *η_yield_*); Bottom all numerical and categorical features (*A_p_*, *A_min_*, *A_max_*, *E_p_*, *E_min_*, *E_max_*, *η_yield_*, *mat0*, *mat1*).

In statistical analysis, it is a common practice to remove outliers since they can skew the results of data analyses and hamper model performance. The outlier removal method employed was the Inter-Quartile Range (IQR) using its default parameters. Data was first divided into quartiles from low to high, first quartile (Q1), the median, and the third quartile (Q3). IQR metric was computed as IQR = Q3 – Q1. Then the upper fence = Q3 + (1.5 * IQR) and the lower fence = Q1 – (1.5 * IQR) were computed. Values that fall outside of the upper and lower fence were considered as outliers and removed. We applied this method to both features under investigation: η_opt_ and PCE. To assess the effect of the outlier removal in the present study, we conducted analyses both with and without outlier removal techniques. The results clearly show a significant reduction in the estimation errors after the IQR-based outlier removal. To build prediction models able to reliably estimate the range of values that were now considered outliers and excluded, we need to provide sufficient samples covering these ranges.


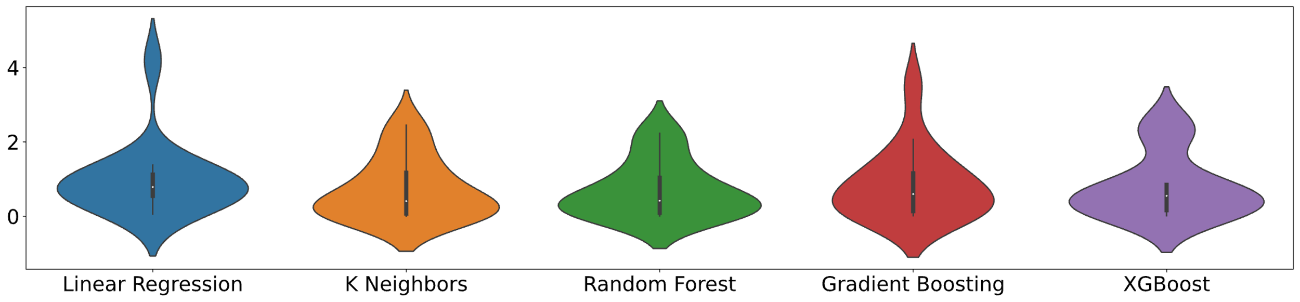


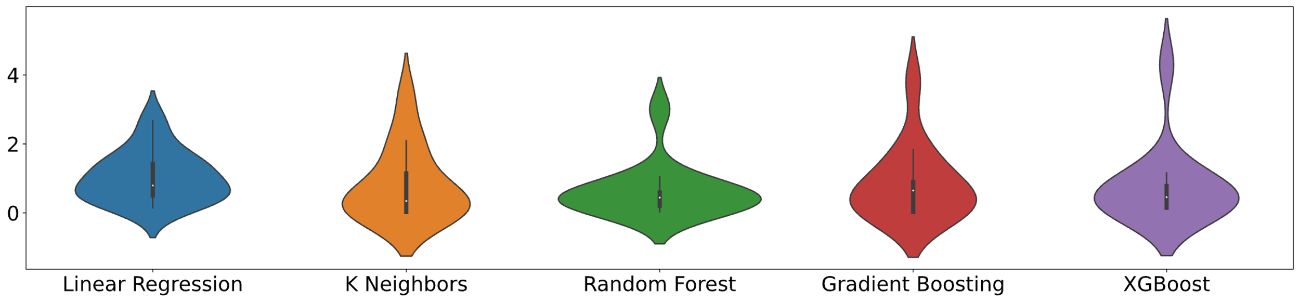


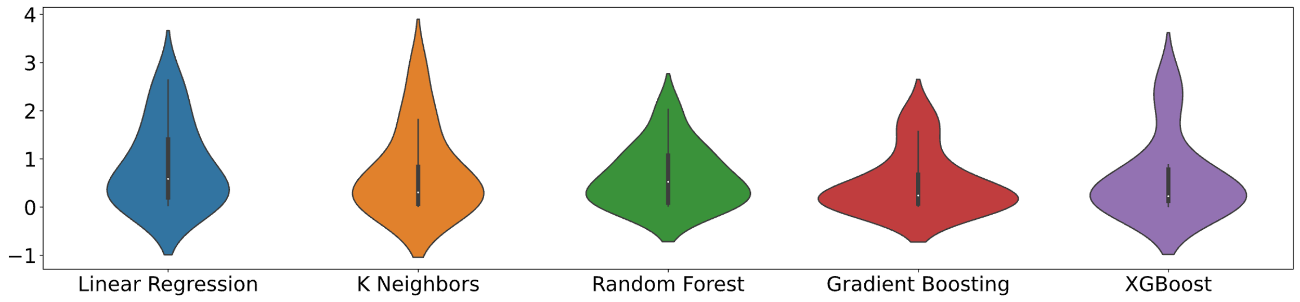


Fig. S4 PCE error distribution for 5 regression models (**data without outliers**). Top (6 input features (*A_p_*, *A_min_*, *A_max_*, *E_p_*, *E_min_*, *E_max_*); Middle (7 input features (*A_p_*, *A_min_*, *A_max_*, *E_p_*, *E_min_*, *E_max_*, *η_yield_*); Bottom all numerical and categorical features (*A_p_*, *A_min_*, *A_max_*, *E_p_*, *E_min_*, *E_max_*, *η_yield_*, *mat0*, *mat1*).


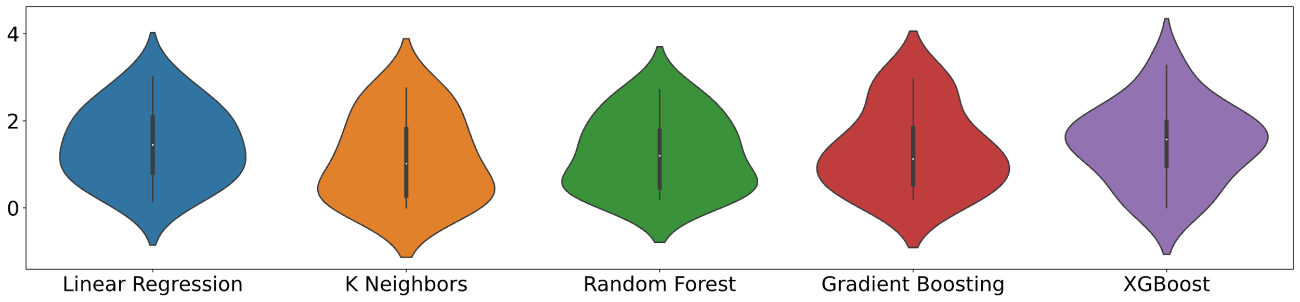


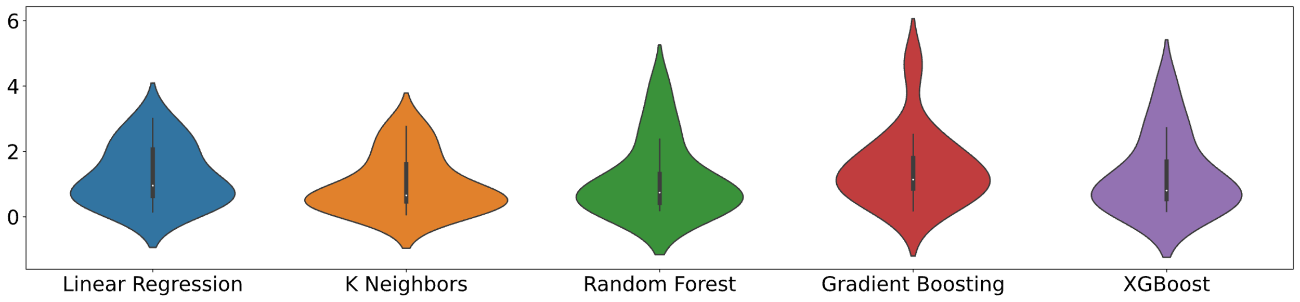


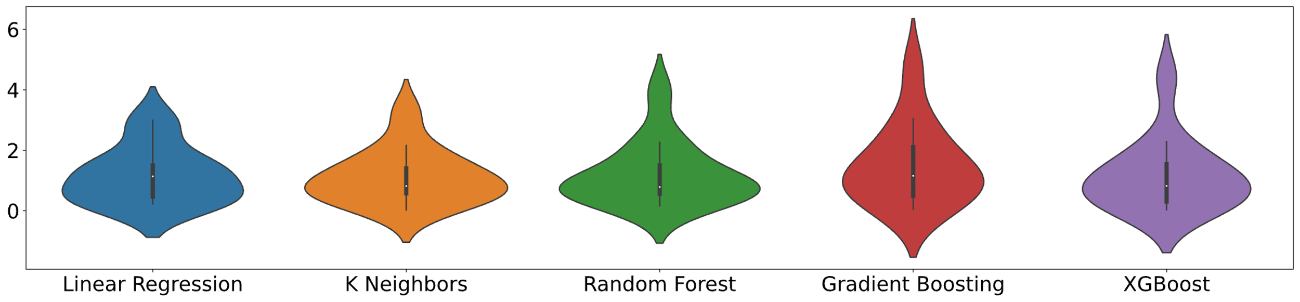


Fig. S5 η_opt_ error distribution for 5 regression models (**data without outliers**). Top (6 input features (*A_p_*, *A_min_*, *A_max_*, *E_p_*, *E_min_*, *E_max_*); Middle (7 input features (*A_p_*, *A_min_*, *A_max_*, *E_p_*, *E_min_*, *E_max_*, *η_yield_*); Bottom all numerical and categorical features (*A_p_*, *A_min_*, *A_max_*, *E_p_*, *E_min_*, *E_max_*, *η_yield_*, *mat0*, *mat1*).

**References**

1. Reisfeld, R., Shamrakov, D. & Jorgensen, C. Photostable solar concentrators based on fluorescent glass-films. *Sol. Energy Mater. Sol. C.* **33**, 417-427 (1994).

2. Sholin, V., Olson, J. D. & Carter, S. A. Semiconducting polymers and quantum dots in luminescent solar concentrators for solar energy harvesting. *J. Appl. Phys.* **101**, 123114 (2007).

3. Misra, V. & Mishra, H. Photoinduced proton transfer coupled with energy transfer: Mechanism of sensitized luminescence of terbium ion by salicylic acid doped in polymer. *J. Chem. Phys.* **128**, 244701 (2008).

4. Nolasco, M. M. *et al.* Engineering highly efficient Eu(III)-based tri-ureasil hybrids toward luminescent solar concentrators. *J. Mater. Chem. A* **1**, 7339-7350 (2013).

5. Graffion, J. *et al.* Modulating the photoluminescence of bridged silsesquioxanes incorporating Eu^3+^-complexed n,n '-diureido-2,2 '-bipyridine isomers: application for luminescent solar concentrators. *Chem. Mater.* **23**, 4773-4782 (2011).

6. Graffion, J. *et al.* Luminescent coatings from bipyridine-based bridged silsesquioxanes containing Eu^3+^ and Tb^3+^ salts. *J. Mater. Chem.* **22**, 13279-13285 (2012).

7. Correia, S. F. H. *et al.* Luminescent solar concentrators: challenges for lanthanide-based organic-inorganic hybrid materials. *J. Mater. Chem. A* **2**, 5580-5596 (2014).

8. Reisfeld, R. New developments in luminescence for solar energy utilization. *Opt. Mater.* **32**, 850-856 (2010).

9. Al-Jarrah, O. Y., Yoo, P. D., Muhaidat, S., Karagiannidis, G. K. & Taha, K. Efficient Machine Learning for Big Data: A Review. *Big Data Res.* **2**, 87-93 (2015).

10. Antony, P. J., Manujesh, P. & Jnanesh, N. A. in *2016 IEEE International Conference on Recent Trends in Electronics, Information & Communication Technology (REICT)*. Bangalore, India. 69-73 (2016).

11. Ferguson, A. L. Machine learning and data science in soft materials engineering. *J. Phys.-Condens. Mat.* **30**, 043002 (2018).
